# *N*′-{2-[2-(3-Methoxyphenyl)ethenyl]phenyl}acetamide

**DOI:** 10.1107/S1600536809017401

**Published:** 2009-05-14

**Authors:** Kartini Ahmad, Noel F. Thomas, Mohd Azlan Nafiah, Khalijah Awang, Seik Weng Ng

**Affiliations:** a Department of Chemistry, University of Malaya, 50603 Kuala Lumpur, Malaysia

## Abstract

In the title compound, C_17_H_17_NO_2_, the phenyl­ene rings are bent with respect to the carbon–carbon double bond [dihedral angle between rings = 39.6 (1)°]. The acetamido group is twisted out of the plane of the aromatic ring [dihedral angle = 44.2 (1)°] in order to form an N–H⋯O hydrogen bond to the acetamido group of an adjacent mol­ecule, generating a zigzag chain running along the *c* axis.

## Related literature

The compound was synthesized in a study on indolostilbenes; see: Ahmad *et al.* (2009 ▶).
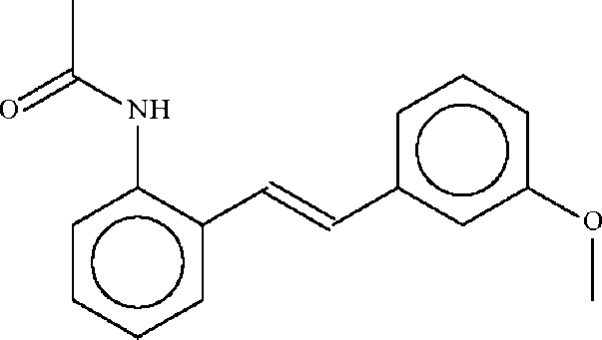


## Experimental

### 

#### Crystal data

C_17_H_17_NO_2_
*M* *_r_* = 267.32
Monoclinic, 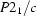

*a* = 14.5588 (5) Å
*b* = 10.3633 (4) Å
*c* = 9.3667 (3) Å
β = 90.118 (1)°
*V* = 1413.22 (9) Å^3^
*Z* = 4
Mo *K*α radiation
μ = 0.08 mm^−1^
*T* = 100 K
0.21 × 0.07 × 0.02 mm

#### Data collection

Bruker SMART APEX diffractometer
Absorption correction: none
12919 measured reflections
3236 independent reflections
2036 reflections with *I* > 2σ(*I*)
*R*_int_ = 0.069

#### Refinement

*R*[*F* ^2^ > 2σ(*F* ^2^)] = 0.052
*wR*(*F* ^2^) = 0.140
*S* = 1.01
3236 reflections
187 parameters
1 restraint
H atoms treated by a mixture of independent and constrained refinement
Δρ_max_ = 0.38 e Å^−3^
Δρ_min_ = −0.25 e Å^−3^

### 

Data collection: *APEX2* software (Bruker, 2008 ▶); cell refinement: *SAINT* (Bruker, 2008 ▶); data reduction: *SAINT*; program(s) used to solve structure: *SHELXS97* (Sheldrick, 2008 ▶); program(s) used to refine structure: *SHELXL97* (Sheldrick, 2008 ▶); molecular graphics: *X-SEED* (Barbour, 2001 ▶); software used to prepare material for publication: *publCIF* (Westrip, 2009 ▶).

## Supplementary Material

Crystal structure: contains datablocks global, I. DOI: 10.1107/S1600536809017401/hg2514sup1.cif

Structure factors: contains datablocks I. DOI: 10.1107/S1600536809017401/hg2514Isup2.hkl

Additional supplementary materials:  crystallographic information; 3D view; checkCIF report

## Figures and Tables

**Table 1:** Hydrogen-bond geometry (Å, °)

*D*—H⋯*A*	*D*—H	H⋯*A*	*D*⋯*A*	*D*—H⋯*A*
N1—H1⋯O1^i^	0.88 (1)	1.93 (1)	2.804 (2)	175 (2)

Symmetry code: (i) 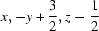.

**Fig. 1.**
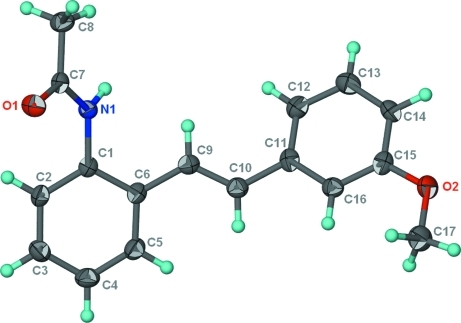
Thermal ellipsoid plot (Barbour, 2001) of C17H17NO2 at the 70% probability level. Hydrogen atoms are drawn as spheres of arbitrary radius.

## supplementary crystallographic information

### Experimental

The compound was synthesized in a study on indolostilbenes (Ahmad *et al.*, 2009). Crystals were grown from its solution in ethyl acetate.

### Refinement

Hydrogen atoms were placed at calculated positions (C–H 0.95–0.98 Å) and were treated as riding on their parent carbon atoms, with *U*(H) set to 1.2–1.5 times *U*~eq~(*C*). The amino H-atom was located in a difference Fourier map, and was refined with a distance restraint of N–H 0.88±0.01 Å; its temperature factor was refined.

### Crystal data

**Table:**

C_17_H_17_NO_2_	*F*(000) = 568
*M**_r_* = 267.32	*D*_x_ = 1.256 Mg m^−^^3^
Monoclinic, *P*2_1_/*c*	Mo *K*α radiation, λ = 0.71073 Å
Hall symbol: -P 2ybc	Cell parameters from 1682 reflections
*a* = 14.5588 (5) Å	θ = 2.4–27.7°
*b* = 10.3633 (4) Å	µ = 0.08 mm^−^^1^
*c* = 9.3667 (3) Å	*T* = 100 K
β = 90.118 (1)°	Plate, colorless
*V* = 1413.22 (9) Å^3^	0.21 × 0.07 × 0.02 mm
*Z* = 4	

### Data collection

**Table:**

Bruker SMART APEX diffractometer	2036 reflections with *I* > 2σ(*I*)
Radiation source: fine-focus sealed tube	*R*_int_ = 0.069
graphite	θ_max_ = 27.5°, θ_min_ = 1.4°
ω scans	*h* = −18→18
12919 measured reflections	*k* = −13→13
3236 independent reflections	*l* = −12→12

### Refinement

**Table:**

Refinement on *F*^2^	Primary atom site location: structure-invariant direct methods
Least-squares matrix: full	Secondary atom site location: difference Fourier map
*R*[*F*^2^ > 2σ(*F*^2^)] = 0.052	Hydrogen site location: inferred from neighbouring sites
*wR*(*F*^2^) = 0.140	H atoms treated by a mixture of independent and constrained refinement
*S* = 1.01	*w* = 1/[σ^2^(*F*_o_^2^) + (0.0657*P*)^2^ + 0.3754*P*] where *P* = (*F*_o_^2^ + 2*F*_c_^2^)/3
3236 reflections	(Δ/σ)_max_ = 0.001
187 parameters	Δρ_max_ = 0.38 e Å^−^^3^
1 restraint	Δρ_min_ = −0.25 e Å^−^^3^

### Fractional atomic coordinates and isotropic or equivalent isotropic displacement parameters (Å^2^)

**Table:**

	*x*	*y*	*z*	*U*_iso_*/*U*_eq_	
O1	0.11400 (10)	0.71029 (14)	0.73510 (14)	0.0246 (4)	
O2	0.65965 (10)	0.58338 (15)	0.10659 (16)	0.0273 (4)	
N1	0.12402 (11)	0.66372 (17)	0.49961 (17)	0.0177 (4)	
H1	0.1185 (16)	0.699 (2)	0.4151 (15)	0.032 (7)*	
C1	0.14037 (13)	0.5285 (2)	0.5123 (2)	0.0172 (4)	
C2	0.08867 (14)	0.4550 (2)	0.6068 (2)	0.0204 (5)	
H2	0.0432	0.4955	0.6639	0.024*	
C3	0.10283 (14)	0.3241 (2)	0.6183 (2)	0.0243 (5)	
H3	0.0686	0.2749	0.6853	0.029*	
C4	0.16751 (15)	0.2639 (2)	0.5315 (2)	0.0269 (5)	
H4	0.1771	0.1734	0.5382	0.032*	
C5	0.21760 (14)	0.3364 (2)	0.4357 (2)	0.0236 (5)	
H5	0.2601	0.2941	0.3746	0.028*	
C6	0.20754 (13)	0.4707 (2)	0.4259 (2)	0.0185 (4)	
C7	0.10934 (13)	0.7448 (2)	0.6095 (2)	0.0187 (4)	
C8	0.08548 (16)	0.8812 (2)	0.5694 (2)	0.0257 (5)	
H8A	0.1255	0.9410	0.6216	0.038*	
H8B	0.0213	0.8985	0.5941	0.038*	
H8C	0.0941	0.8931	0.4665	0.038*	
C9	0.26855 (14)	0.5474 (2)	0.3343 (2)	0.0208 (5)	
H9	0.2507	0.6338	0.3145	0.025*	
C10	0.34701 (14)	0.5057 (2)	0.2767 (2)	0.0222 (5)	
H10	0.3623	0.4177	0.2919	0.027*	
C11	0.41182 (14)	0.5822 (2)	0.1925 (2)	0.0206 (5)	
C12	0.38525 (15)	0.6915 (2)	0.1148 (2)	0.0242 (5)	
H12	0.3230	0.7191	0.1156	0.029*	
C13	0.45036 (16)	0.7595 (2)	0.0361 (2)	0.0261 (5)	
H13	0.4322	0.8334	−0.0168	0.031*	
C14	0.54070 (15)	0.7208 (2)	0.0343 (2)	0.0223 (5)	
H14	0.5844	0.7671	−0.0208	0.027*	
C15	0.56799 (14)	0.6138 (2)	0.1129 (2)	0.0218 (5)	
C16	0.50366 (14)	0.5444 (2)	0.1902 (2)	0.0212 (5)	
H16	0.5224	0.4701	0.2421	0.025*	
C17	0.69172 (16)	0.4821 (2)	0.1966 (2)	0.0302 (5)	
H17A	0.7586	0.4749	0.1882	0.045*	
H17B	0.6631	0.4005	0.1677	0.045*	
H17C	0.6755	0.5012	0.2959	0.045*	

### Atomic displacement parameters (Å^2^)

**Table:**

	*U*^11^	*U*^22^	*U*^33^	*U*^12^	*U*^13^	*U*^23^
O1	0.0362 (9)	0.0232 (8)	0.0144 (7)	0.0009 (7)	0.0007 (6)	−0.0012 (6)
O2	0.0182 (8)	0.0272 (9)	0.0366 (9)	−0.0019 (6)	0.0023 (6)	0.0044 (7)
N1	0.0207 (9)	0.0181 (9)	0.0144 (8)	0.0017 (7)	0.0011 (7)	0.0019 (7)
C1	0.0157 (10)	0.0204 (11)	0.0156 (9)	0.0000 (8)	−0.0024 (8)	−0.0020 (8)
C2	0.0162 (10)	0.0245 (11)	0.0203 (10)	0.0016 (9)	0.0011 (8)	0.0000 (9)
C3	0.0232 (12)	0.0231 (12)	0.0266 (11)	−0.0028 (9)	0.0005 (9)	0.0043 (9)
C4	0.0252 (12)	0.0170 (11)	0.0387 (13)	−0.0006 (9)	−0.0006 (10)	−0.0002 (10)
C5	0.0186 (11)	0.0227 (12)	0.0296 (11)	0.0013 (9)	0.0017 (9)	−0.0028 (10)
C6	0.0150 (10)	0.0210 (11)	0.0195 (10)	−0.0009 (8)	−0.0022 (8)	−0.0026 (9)
C7	0.0150 (10)	0.0214 (11)	0.0196 (10)	−0.0007 (8)	0.0010 (8)	−0.0007 (8)
C8	0.0349 (13)	0.0208 (12)	0.0213 (10)	0.0048 (9)	0.0023 (9)	−0.0014 (9)
C9	0.0212 (11)	0.0190 (11)	0.0220 (10)	0.0008 (9)	−0.0011 (8)	−0.0008 (9)
C10	0.0247 (12)	0.0197 (11)	0.0222 (10)	0.0001 (9)	0.0010 (9)	−0.0009 (9)
C11	0.0225 (11)	0.0233 (11)	0.0160 (9)	−0.0030 (9)	0.0018 (8)	−0.0051 (9)
C12	0.0220 (11)	0.0268 (12)	0.0238 (11)	0.0020 (9)	−0.0007 (9)	−0.0017 (10)
C13	0.0313 (13)	0.0237 (12)	0.0234 (11)	−0.0005 (10)	−0.0024 (9)	0.0028 (9)
C14	0.0241 (12)	0.0215 (11)	0.0213 (10)	−0.0055 (9)	0.0013 (8)	0.0016 (9)
C15	0.0182 (11)	0.0254 (12)	0.0218 (10)	−0.0016 (9)	−0.0005 (8)	−0.0055 (9)
C16	0.0238 (11)	0.0194 (11)	0.0203 (10)	0.0004 (9)	−0.0001 (8)	−0.0007 (9)
C17	0.0205 (12)	0.0338 (14)	0.0361 (13)	−0.0009 (10)	−0.0031 (10)	0.0052 (11)

### Geometric parameters (Å, °)

**Table:**

O1—C7	1.231 (2)	C8—H8B	0.9800
O2—C15	1.373 (2)	C8—H8C	0.9800
O2—C17	1.424 (3)	C9—C10	1.336 (3)
N1—C7	1.346 (3)	C9—H9	0.9500
N1—C1	1.426 (3)	C10—C11	1.464 (3)
N1—H1	0.877 (10)	C10—H10	0.9500
C1—C2	1.391 (3)	C11—C16	1.394 (3)
C1—C6	1.405 (3)	C11—C12	1.400 (3)
C2—C3	1.376 (3)	C12—C13	1.393 (3)
C2—H2	0.9500	C12—H12	0.9500
C3—C4	1.393 (3)	C13—C14	1.375 (3)
C3—H3	0.9500	C13—H13	0.9500
C4—C5	1.380 (3)	C14—C15	1.388 (3)
C4—H4	0.9500	C14—H14	0.9500
C5—C6	1.402 (3)	C15—C16	1.386 (3)
C5—H5	0.9500	C16—H16	0.9500
C6—C9	1.470 (3)	C17—H17A	0.9800
C7—C8	1.504 (3)	C17—H17B	0.9800
C8—H8A	0.9800	C17—H17C	0.9800
			
C15—O2—C17	117.48 (17)	C10—C9—C6	125.4 (2)
C7—N1—C1	125.17 (17)	C10—C9—H9	117.3
C7—N1—H1	114.4 (16)	C6—C9—H9	117.3
C1—N1—H1	120.2 (16)	C9—C10—C11	126.5 (2)
C2—C1—C6	120.73 (19)	C9—C10—H10	116.7
C2—C1—N1	120.06 (18)	C11—C10—H10	116.7
C6—C1—N1	119.19 (18)	C16—C11—C12	118.93 (19)
C3—C2—C1	120.6 (2)	C16—C11—C10	118.36 (19)
C3—C2—H2	119.7	C12—C11—C10	122.71 (19)
C1—C2—H2	119.7	C13—C12—C11	119.8 (2)
C2—C3—C4	119.8 (2)	C13—C12—H12	120.1
C2—C3—H3	120.1	C11—C12—H12	120.1
C4—C3—H3	120.1	C14—C13—C12	120.7 (2)
C5—C4—C3	119.6 (2)	C14—C13—H13	119.6
C5—C4—H4	120.2	C12—C13—H13	119.6
C3—C4—H4	120.2	C13—C14—C15	119.9 (2)
C4—C5—C6	121.8 (2)	C13—C14—H14	120.0
C4—C5—H5	119.1	C15—C14—H14	120.0
C6—C5—H5	119.1	O2—C15—C16	124.12 (19)
C5—C6—C1	117.31 (19)	O2—C15—C14	115.96 (18)
C5—C6—C9	120.78 (19)	C16—C15—C14	119.9 (2)
C1—C6—C9	121.83 (19)	C15—C16—C11	120.7 (2)
O1—C7—N1	122.77 (19)	C15—C16—H16	119.6
O1—C7—C8	121.59 (18)	C11—C16—H16	119.6
N1—C7—C8	115.64 (17)	O2—C17—H17A	109.5
C7—C8—H8A	109.5	O2—C17—H17B	109.5
C7—C8—H8B	109.5	H17A—C17—H17B	109.5
H8A—C8—H8B	109.5	O2—C17—H17C	109.5
C7—C8—H8C	109.5	H17A—C17—H17C	109.5
H8A—C8—H8C	109.5	H17B—C17—H17C	109.5
H8B—C8—H8C	109.5		
			
C7—N1—C1—C2	42.6 (3)	C1—C6—C9—C10	162.9 (2)
C7—N1—C1—C6	−138.9 (2)	C6—C9—C10—C11	−175.99 (18)
C6—C1—C2—C3	0.5 (3)	C9—C10—C11—C16	154.0 (2)
N1—C1—C2—C3	178.97 (18)	C9—C10—C11—C12	−25.9 (3)
C1—C2—C3—C4	−2.0 (3)	C16—C11—C12—C13	0.5 (3)
C2—C3—C4—C5	0.7 (3)	C10—C11—C12—C13	−179.63 (19)
C3—C4—C5—C6	2.1 (3)	C11—C12—C13—C14	−0.2 (3)
C4—C5—C6—C1	−3.5 (3)	C12—C13—C14—C15	−0.9 (3)
C4—C5—C6—C9	173.33 (19)	C17—O2—C15—C16	−6.6 (3)
C2—C1—C6—C5	2.2 (3)	C17—O2—C15—C14	174.07 (18)
N1—C1—C6—C5	−176.28 (18)	C13—C14—C15—O2	−179.00 (19)
C2—C1—C6—C9	−174.62 (18)	C13—C14—C15—C16	1.7 (3)
N1—C1—C6—C9	6.9 (3)	O2—C15—C16—C11	179.34 (19)
C1—N1—C7—O1	4.4 (3)	C14—C15—C16—C11	−1.4 (3)
C1—N1—C7—C8	−174.86 (18)	C12—C11—C16—C15	0.3 (3)
C5—C6—C9—C10	−13.8 (3)	C10—C11—C16—C15	−179.58 (18)

### Hydrogen-bond geometry (Å, °)

**Table:**

*D*—H···*A*	*D*—H	H···*A*	*D*···*A*	*D*—H···*A*
N1—H1···O1^i^	0.88 (1)	1.93 (1)	2.804 (2)	175 (2)

Symmetry codes: (i) *x*, −*y*+3/2, *z*−1/2.
